# The effectiveness of 6-week superimposed neuromuscular electrical stimulation in knee osteoarthritis: an assessor blinded randomized controlled clinical trial

**DOI:** 10.1007/s00402-025-05958-x

**Published:** 2025-06-21

**Authors:** Oguzhan Mete, Ayşegül Yaman, Çiğdem Zaman, Soneser Kara, Mustafa Ertuğrul Yaşa, Selman Aktaş, Emre Adıgüzel

**Affiliations:** 1 University of Health Sciences, Ankara, Turkey; 2 Ankara Etlik City Hospital, Ankara, Turkey; 3 Ankara Etlik City Hospital, Ankara, Turkey; 4 Ankara Bilkent City Hospital, Ankara, Turkey; 5 University of Health Sciences, İstanbul, Turkey

**Keywords:** Knee osteoarthritis, Electrical stimulation therapy, Quadriceps muscles, Physical therapy modalities

## Abstract

**Introduction:**

Conservative treatment of knee osteoarthritis (KOA) is crucial for alleviating patient complaints and postponing disability from the onset of symptoms until the disease progresses to the point where surgery is inevitable. Although as a part of conservative management, Superimposed Neuromuscular Electrical Stimulation (sNMES), which combines electrical stimulation with voluntary contraction, has gained popularity in clinical practice, the effects of sNMES in KOA are unknown. Therefore, the study aimed to investigate the effects of sNMES in patients with KOA.

**Materials and methods:**

Fifty-eight participants with KOA were randomly divided into three groups (sNMES + CP (conventional physiotherapy), NMES (Neuromuscular Electrical Stimulation) + CP, and only CP). The treatments lasted 3 times for 6 weeks. Before and after treatments, the quadriceps and femoral cartilage thickness, sensorimotor function (balance and proprioception), physical function (quadriceps muscle strength, knee range of motion, and 30-second sit-to-stand test), and functional status (Short Form-36 (SF36) and Western Ontario and McMaster Universities Osteoarthritis Index (WOMAC)) were evaluated.

**Results:**

Regarding timeXgroup effect when comparing groups, the sNMES + CP showed significant improvements over NMES + CP and only CP in quadriceps muscle strength (*p* < 0.01), SF36_Energy (*p* < 0.01), and SF36_Social Functioning (*p* < 0.01). Both the sNMES + CP (*p* < 0.01) and only CP groups (*p* < 0.01) showed significant changes in SF36_Pain scores, while all groups had significant reductions in WOMAC Scores (*p* < 0.05), with the sNMES + CP group showing the greatest improvement (*p* < 0.01). No adverse effects were reported in all treatment groups.

**Conclusion:**

sNMES + CP proved more effective than NMES + CP or only CP in enhancing quadriceps muscle strength and improving functional status. Incorporating sNMES instead of NMES into CP could be a more effective intervention for treating KOA, particularly when the goal is to enhance quadriceps strength, thereby improving functional status. In conservative management of KOA, collaboration between orthopedists and physiatrists is crucial for addressing functional improvement regarding the effect of sNMES.

## Introduction

Osteoarthritis (OA) is a common condition seen in orthopedic clinics that leads to long-term disability and negatively impacts the quality of life (QoL) of those affected [1]. The knee joint is the most impacted by OA, mainly because of its essential function as a weight-bearing joint and its exposure to considerable mechanical stress during everyday activities. Knee osteoarthritis (KOA) represents an important global health burden due to its high prevalence, chronic nature, and substantial impact on QoL [1, 2]. Beyond its substantial economic burden, KOA profoundly affects physical function, limiting mobility and independence, which can lead to social isolation and psychological distress [3]. This underscores the critical importance of implementing preventive measures, early diagnosis, and employing effective management strategies to mitigate both individual and societal impact.

Currently, there exists no definitive cure for OA, and the available treatment options are notably restricted. Non-surgical treatments are crucial for alleviating patient complaints and postponing disability during the period from the onset of symptoms until the disease progresses to the point where surgery is inevitable. Intra-articular injections and physiotherapy are key elements of conservative treatments [4]. Intra-articular injections provide effective immediate pain relief, while physiotherapy is essential for maintaining joint function, particularly in long-term management. The main components of physiotherapy include therapeutic exercises, electrotherapy, manual therapy, and an emerging focus on tele-rehabilitation [4–6]. Exercise plays a central role in managing KOA. Specifically strengthening exercise for the quadriceps muscle, which is the main shock absorber of the knee joint, is crucial as quadriceps weakness in KOA is determined to be related to disability level and pain [7]. Painful pathologies like KOA are often associated with a decrease in neural drive to surrounding muscles, particularly the quadriceps [8, 9]. Given these challenges, integrating external electrical inputs, such as neuromuscular electrical stimulation (NMES), into rehabilitation programs can help overcome neural inhibition and enhance muscle activation. The physiological effects of each stimulus may compound even when electrical stimulation is applied in conjunction with exercise, a practice known as superimposed NMES (sNMES) [10]. NMES causes muscle contractions by involuntarily activating the muscles, while sNMES involves a combination of active and passive stimulation, encouraging patients to perform active contractions alongside the stimulation provided by NMES.

sNMES for knee-related disorders and its adoption instead of NMES has become widespread [11]. However, to the best of our knowledge, no study has been conducted to investigate the effectiveness of sNMES in KOA. The current study aimed to investigate the effects of sNMES in patients with KOA. Therefore, we compared the effects of three different rehabilitation programs (conventional physiotherapy (CP), NMES + CP, and sNMES + CP) on the quadriceps and femoral cartilage thickness, sensorimotor and physical function, and self-reported functional status in patients with KOA.

## Materials and methods

### Design and participants

This prospective, randomized controlled single-blinded clinical trial was conducted at the Ankara Etlik City Hospital Physical Medicine and Rehabilitation Hospital between October 2023 and December 2024. The study design was registered at http://clinicaltrials.gov (NCT06277570) and approved by the Ankara Etlik City Hospital Clinical Research Ethics Committee (AESH-EK1-2023-487). The research adhered to the principles outlined in the Declaration of Helsinki, and written informed consent was obtained from all participants.

Patients aged between 45 and 65 years, diagnosed with KOA according to the American College of Rheumatology criteria, and classified as grade 2 or 3 based on the Kellgren-Lawrence score [12], were invited. The exclusion criteria of the study were determined as: (1) OA in the hip, ankle, or foot joints. (2) History of knee surgery (e.g., arthroplasty, meniscectomy). (3) Active knee joint synovitis. (4) Current knee-related pathologies (e.g., meniscus tears, sprain). (5) Deformity in the lower back, leg, hip, knee, or ankle. (6) Diseases hindering clinical assessments (e.g., inflammatory rheumatic diseases, neurological issues). (7) Conditions contraindicating electrotherapy (e.g., malignancy, pacemaker). (8) Conditions limiting exercise (e.g., orthopedic, cardiopulmonary diseases). (9) Received physiotherapy in the last three months. (10) Did not volunteer to participate. Participants were excluded from the study if they did not attend treatment regularly or discontinued it for personal reasons. The use of medications that could affect the study results was restricted throughout the study period.

### Interventions

Participants who met the criteria were randomly divided into three groups (sNMES + CP, NMES + CP, and only CP). Each participant received 18 sessions of treatment, occurring three times a week.

### Conventional physiotherapy

The CP for all KOA patients in our clinic consisted of thermotherapy (using hot packs), electrotherapy (including TENS (Transcutaneous electrical nerve stimulation) and therapeutic ultrasound), and a supervised group exercise program. The CP began by applying a hot pack to the knee region for 20 min, followed by TENS. The conventional form of TENS was utilized, with parameters set to symmetrical biphasic pulsed current at a frequency of 100 Hz and a pulse duration of 100 microseconds. Subsequently, continuous ultrasound was applied at a frequency of 1 MHz and an intensity of 1.5 W/cm² for 8 min. After completing the thermotherapy and electrotherapy, participants participated in a supervised group exercise program, which included strengthening and stretching exercises for the knee and hip muscles [13].

### NMES protocol

The NMES was applied with the BTL^®^ 4000 electrotherapy device (BTL Industries, Stevenage, Hertfordshire, UK) with the listed parameters: waveform: pulsed symmetrical biphasic rectangular, frequency: 50 Hz, pulse duration: 300 µs, duty cycle: 1/2 (10 s stimulation, 20 s rest), ramp up/down: 1/1 second, time: 20 min (40 times contraction), amplitude: from visible contraction to maximally tolerated. Participants were positioned in a long sitting posture, supported by a thin towel under the knee. Adhesive electrodes (5 cm x 5 cm) were positioned distally vastus medialis (VM) and proximally vastus lateralis (VL), in line with the quadriceps motor points. The stimulation amplitude was adjusted for each participant [14].

### Superimposed NMES

**Fig. 1:**
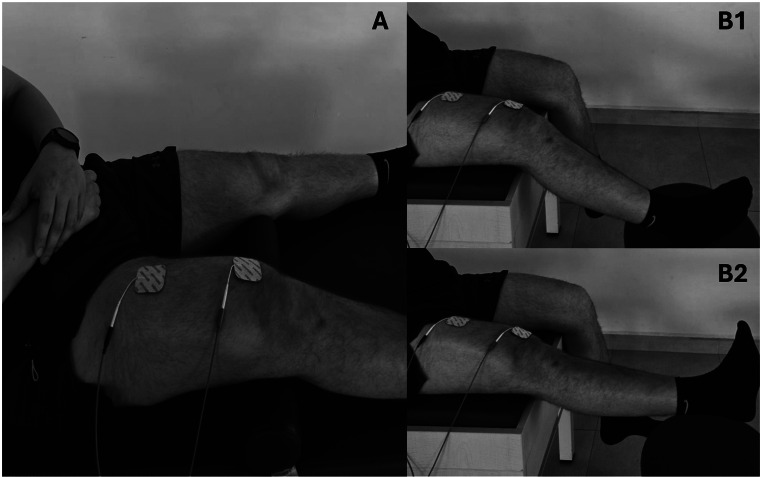
Exercises with superimposed NMES

The same parameters and treatment sessions used for NMES were applied to sNMES. Unlike NMES, participants were instructed to perform voluntary contractions with each stimulation. Voluntary contraction was applied during two different exercises: isometric quadriceps strengthening and 0–30-degree terminal knee extension. Participants were instructed to contract their quadriceps muscles in conjunction with the NMES following its stimulation/rest cycle. Each exercise was performed for 10 min, totaling 20 min of NMES, with 20 repetitions of isometric and 20 repetitions of isotonic contractions of quadriceps [14] (Fig. 1).

### Outcomes

The demographic and physical specifications (age, sex, body mass index (BMI)) of the participants were recorded. The clinical history (presence of exclusion criteria, presence of chronic metabolic disease, symptomatic knee, and resting and activity pain intensity according to the Visual Analog Scale [15]) was examined and recorded. Before and after treatment, the quadriceps and femoral cartilage thickness, sensorimotor function, physical function (quadriceps muscle strength, knee active range of motion (AROM), 30-second sit-to-stand test, and self-reported functional status were evaluated.

### Quadriceps and femoral cartilage thickness

A physiatrist experienced in musculoskeletal ultrasound imaging techniques evaluated the quadriceps muscle thickness and femoral knee cartilage using a diagnostic ultrasound device (Logiq P9, GE Medical Systems, USA) with a 4.0 MHz − 15.0 MHz linear probe (GE ML6-15).

**Fig. 2:**
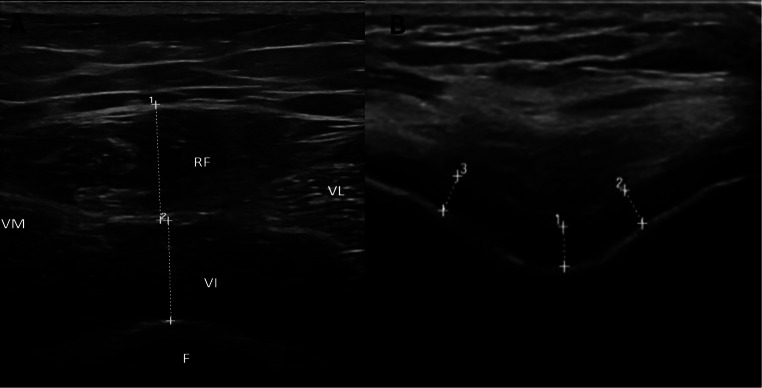
Ultrasonographic measurement of quadriceps and femoral cartilage thickness

The thickness of the quadriceps muscle (rectus femoris (RF), VM, intermedius (VI), and VL) was evaluated. Thickness measurements for each muscle were taken at the midpoint of the thigh, with measurement points identified by measuring from the lateral condyle of the femur to the greater trochanter. The marked measurement sites were obtained and assessed while the participants were positioned supine. Each measurement was performed twice, and the average of the two was recorded for the thickness of the RF, VM, VI, and VL, expressed in centimeters (Fig. 2) [16].

Femoral knee cartilage thickness was assessed while participants laying supine, and their knees were maximally hyperflexed. The probe was positioned axially in the suprapatellar region. Measurements were taken from each knee joint at three specific locations: the medial condyle, lateral condyle, and intercondylar area (Fig. 2). The mean value of the three measurements at each site was recorded [17].

### Sensorimotor function

Balance and proprioception, the main components of sensorimotor function, were assessed with the Balance Error Scoring System (BESS) and knee joint position sense (JPS), respectively. The BESS consists of six testing conditions, which include three 20-second standardized stances: double-leg, single, and tandem. All parameters of the BESS exhibited excellent reliability in assessing balance in the KOA, with an inter-intraclass coefficient greater than 0.90 [18]. These stances are performed on firm and foam surfaces, with participants closing their eyes and hands on their hips. An error is defined as any of the following actions: opening the eyes, lifting hands off the hips, stepping, stumbling, or falling out of position, lifting the forefoot or heel, abducting the hip by more than 30 degrees, or failing to return to the test position within 5 s. The test was conducted with bare feet. Before the test, the procedure was explained, and the testing was carried out in the order of double-leg, single-leg, and tandem stances. Error scores were recorded for each stance as a total [18].

The knee JPS was evaluated using a digital inclinometer. It was found reliable and valid to evaluate the JPS in KOA [19]. The inclinometer was attached to the posterior aspect of the mid-calf. Participants were placed in a supine position, and their knees were passively moved from a neutral position to the targeted angles of 30° of flexion. They were instructed to memorize these angles. Afterward, the participants returned to the neutral position and were asked to return their knees to the memorized position. The Joint Position Error (JPE), which measures the degree of deviation from the targeted angle, was recorded. Each test was conducted three times at 20-second intervals, and the average score was used for analysis. A higher JPE score indicates a weaker JPS [19].

### Physical function

The physical function assessments included quadriceps muscle strength, AROM, and 30-second sit-to-stand test. The quadriceps muscle strength was evaluated using a handheld dynamometer, which proved reliable for assessing knee muscle strength [20]. Participants were seated with their feet and hands suspended and their knees flexed at 60 degrees. The assessor positioned himself in a squat with their back against the wall for support, extending both arms toward the patient’s dominant leg for stabilization. The handheld dynamometer was placed on the anterior aspect of the participant’s leg, three fingers above the distal part of the medial malleolus. Participants were instructed to perform maximum knee extension strength for five seconds. Two trials were conducted with a one-minute rest period between measurements to minimize fatigue. The maximum value from the two trials was used for analysis [20].

Knee joint sagittal plane AROM was evaluated using a standard goniometer, which is a valid and reliable tool for assessing knee AROM [21]. The participants were placed in a prone position to assess AROM in knee flexion and hyperextension. The pivot point of the goniometer was positioned at the lateral condyle of the femur, with the fixed arm aligned along the lateral aspect of the femur. During the measurement of AROM, the goniometer’s mobile arm followed the movement of the fibula. The measurement was finalized when the participant reached the maximum flexion angle. The average of three measurements was then calculated and recorded [21].

The 30-second sit-to-stand test was used to evaluate lower extremity physical function. This method has proven to be a reliable tool for assessing this function in KOA [22]. For the test, a chair that is 45 centimeters high without arm support was used. Participants started sitting on the chair with their arms folded across their chests. They were instructed to stand up and sit back down as quickly as possible. A stand was counted only if the participant stood fully (with straight hips and knees) and returned to the sitting position. The total number of stands completed in 30 s was recorded [22].

### Self-reported functional status

Participants’ disease-specific functional status were assessed with the Short Form-36 (SF36) and Western Ontario and McMaster Universities Osteoarthritis Index (WOMAC) which are patient-reported questionnaires. SF36 consists of 36 questions with 8 subscales: physical function, role limitations due to physical and emotional health, energy/fatigue, mental health, social functioning, bodily pain, and general health perception. Each category is scored independently, with the lowest possible score being 0 and the highest score being 100. Higher scores reflect an improved functional status [23].

Disease-specific functional status refers to disability evaluated using the WOMAC, which contains 24 questions in three categories (pain, stiffness, and physical function). This assessment employs a 5-point Likert scale, ranging from 0 to 4, where 0 indicates none and 4 indicates extreme. The total score is calculated by summing up all items. The total score was recorded. A high score on the WOMAC scale suggests low functional status and high levels of disability [24].

### Randomization and blinding

A simple randomization method was employed. Before participant recruitment, a randomization list was created by an individual who was not involved in any clinical investigations. Each participant was assigned a numeric code. Potential participants were invited in groups based on their positions on the waiting list. The distribution of these groups was determined sequentially before sending out the invitations, and this process was kept confidential from the clinical assessors.

The researchers administering the treatment were aware of which study arm the patients were assigned to; however, this information was concealed from the researchers who collected the outcome data. Therefore, the assessors of the study were blinded to the treatment allocation. Additionally, the statistical analysis was conducted by a statistician who did not participate in the clinical process of the study. The intervention arms were randomly labeled as A, B, and C in the data set, ensuring that the statistician remained unaware of the patients’ group assignments.

### Statistical analysis

The required sample size was determined by using G*Power version 3.1 software, based on the thickness of the RF. Four data from each group, which were selected randomly, were used for calculations. After calculations, we found that a sample of at least eighty-one participants (seventeen per group) would be needed to obtain 80% power with an F = 0.45 effect size, α = 0.05 type I error, and β = 0.20 type II error.

Data was analyzed using SPSS version 25 (IBM Corp., 2017, IBM SPSS Statistics for Windows, version 25.0, Armonk, NY: IBM Corp). Numerical variables were presented as mean ± standard deviation, and categorical variables as numbers (percentage). The distribution of the variables was analyzed with the Shapiro-Wilk test. Group comparisons of numerical variables identified as normally distributed were conducted using one-way analysis of variance (ANOVA). The Fisher’s Exact Test was performed to analyze categorical variables. Repeated measures ANOVA was conducted to assess group effects over time (pre-test vs. post-test), assuming normality. Post-hoc comparisons with Bonferroni correction were applied in the presence of significant main effects or interactions. A confidence level of 95% was maintained for all analyses. A p-value of less than 0.05 was considered statistically significant.

## Results

**Fig. 3:**
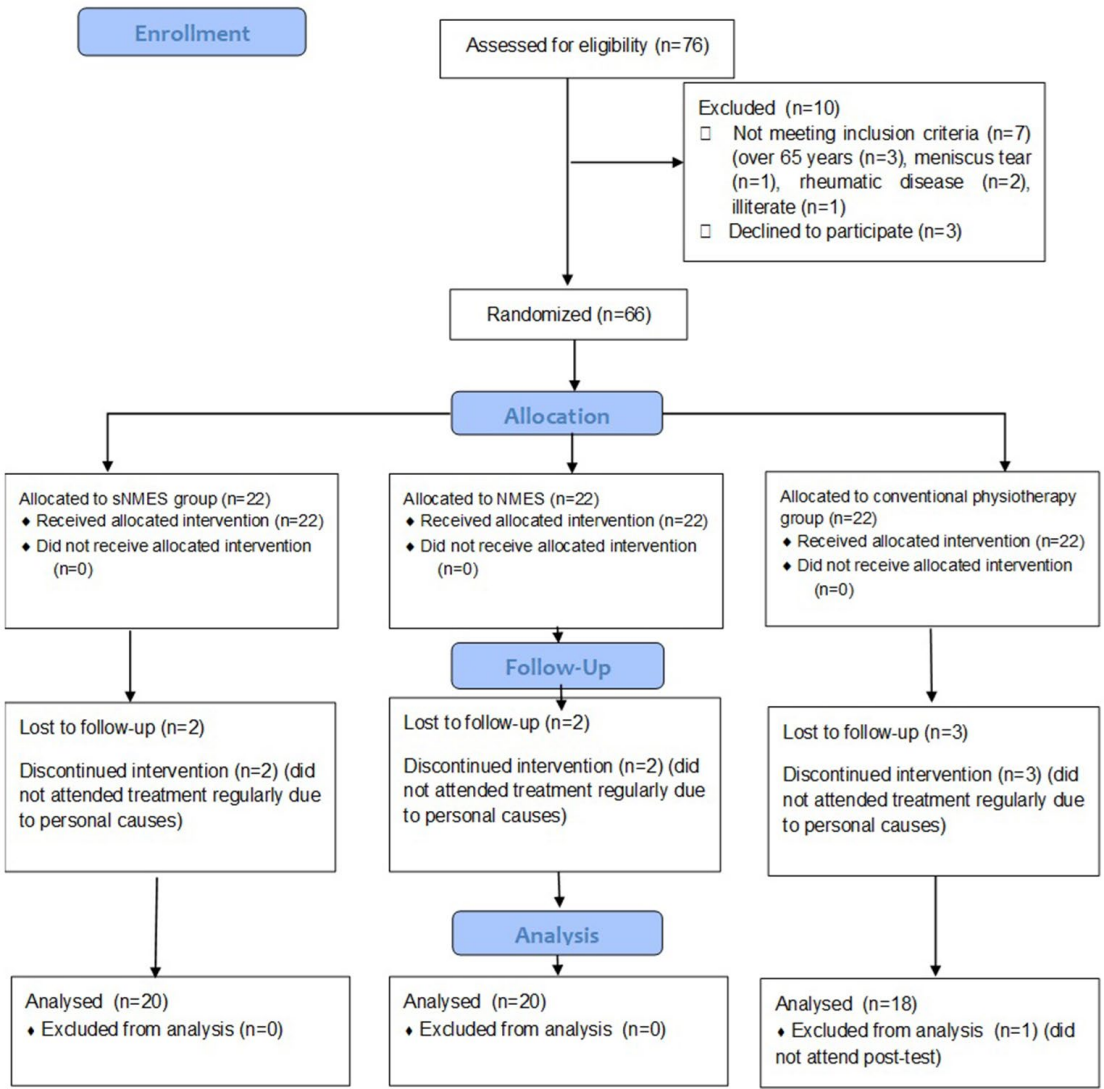
The flowchart of the study

**Fig. 4:**
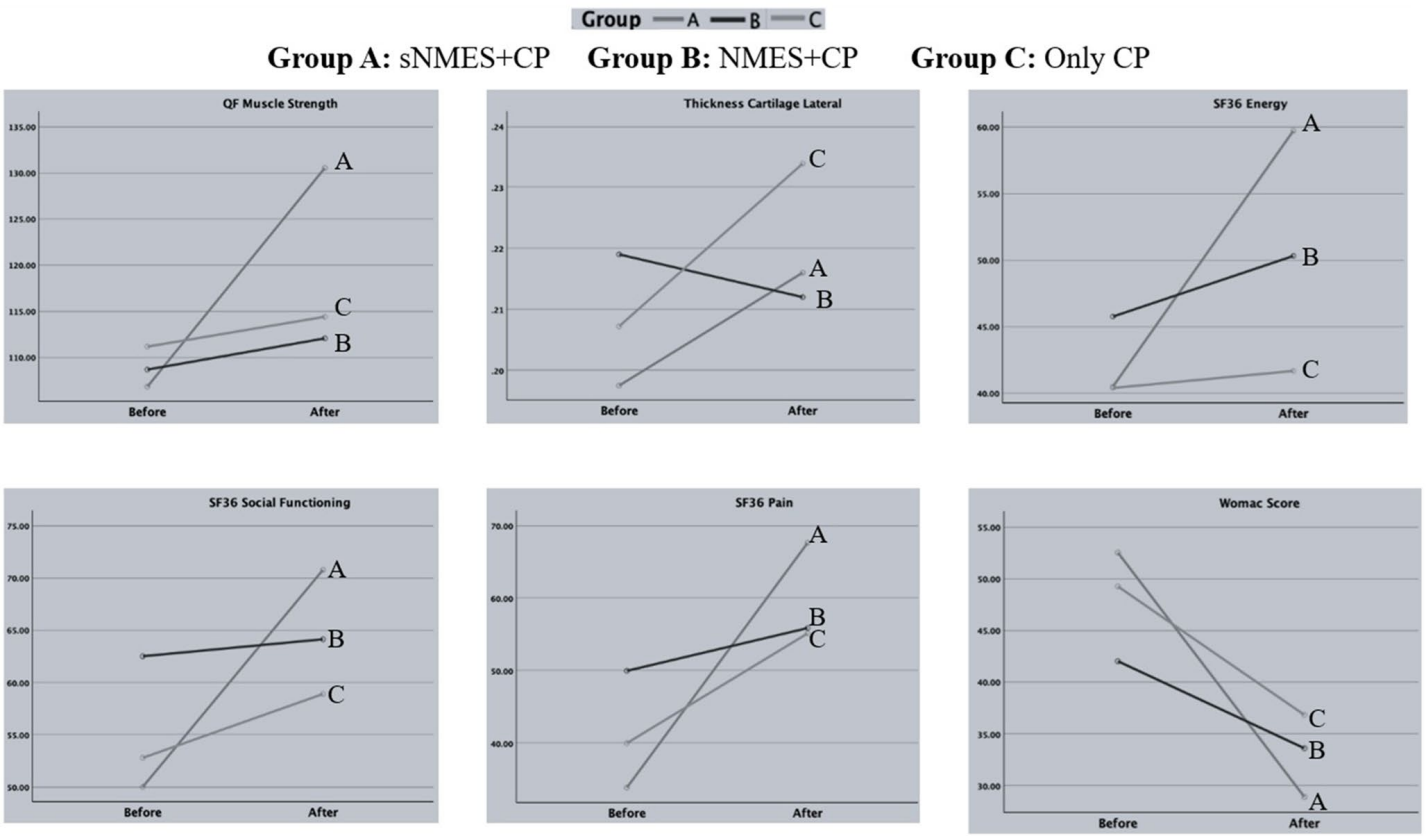
Comparison between groups of variables found to be significant in terms of Group X Time interactions. sNMES: Superimposed Neuromuscular Electrical Stimulation, NMES: Neuromuscular Electrical Stimulation, CP: Conventional physiotherapy, SF36: Short Form-36, Womac: Western Ontario and McMaster Universities Osteoarthritis Index, QF: Quadriceps

The enrollment, allocation, follow-up, and final analysis process are summarized in Fig. 3. The age, BMI, and gender were similar between groups (*p* > 0.05). The baseline resting and activity pain intensity was similar between groups (*p* > 0.05) (Table 1). The descriptive values of outcome variables are provided in Table 2. The effect of time on the groups is analyzed in Table 3. For the variables that were found to be significant, we examined the changes within the groups before and after treatment by conducting multiple comparisons. The results of these comparisons are presented in Table 4; Fig. 4. No adverse effects were reported in all groups.

**Table 1:** Comparison of the demographic and physical characteristics of groups

	sNMES + CP (*n*:20)	NMES + CP (*n*:20)	Only CP (*n*:18)	*p*
Age (years)	58.65 ± 4.42	59.15 ± 5.37	58.16 ± 4.90	0.828^a^
BMI (kg/m^2^)	30.01 ± 4.66	32.63 ± 10.65	31.76 ± 6.35	0.553^a^
Sex (n (%))				
Female	18 (90)	16 (80)	16 (88.9)	0.607^b^
Male	2 (10)	4 (20)	2 (11.1)	
Pain Intensity (VAS, cm)				
Resting	4.54 ± 3.16	3.54 ± 2.61	4.32 ± 3.23	0.549^a^
Activity	7.16 ± 2.20	6.65 ± 2.51	6.89 ± 2.20	0.707^a^

n: Number, kg/m^2^: kilogram/meter square, n: Number, VAS: Visual Analog Scale, cm: Centimeter, sNMES: Superimposed Neuromuscular Electrical Stimulation, NMES: Neuromuscular Electrical Stimulation, CP: Conventional physiotherapy,

^a^One-way ANOVA, ^b^Fisher’s Exact Test

**Table 2:** Descriptive values of outcomes

	sNMES + CP Group		NMES + CP Group		Only CP Group	
	Before	After	Before	After	Before	After
Muscle and Cartilage Morphology						
Thickness M. Rectus femoris	1.2 ± 0.3	1.3 ± 0.2	1.3 ± 0.2	1.4 ± 0.2	1.3 ± 0.3	1.3 ± 0.2
Thickness M. Vastus intermedius	1.0 ± 0.4	1.1 ± 0.3	1.0 ± 0.3	1.1 ± 0.3	1.1 ± 0.3	1.1 ± 0.3
Thickness M. Vastus lateralis	2.0 ± 0.6	2.3 ± 0.7	2.5 ± 0.6	2.7 ± 0.6	2.5 ± 0.6	2.5 ± 0.6
Thickness M. Vastus medialis	2.2 ± 0.6	2.3 ± 0.6	2.3 ± 0.4	2.4 ± 0.4	2.7 ± 1.7	2.9 ± 2.0
Thickness cartilage medial	0.2 ± 0.0	0.2 ± 0.0	0.2 ± 0.0	0.2 ± 0.0	0.2 ± 0.0	0.2 ± 0.0
Thickness cartilage lateral	0.1 ± 0.0	0.2 ± 0.0	0.2 ± 0.0	0.2 ± 0.0	0.2 ± 0.0	0.2 ± 0.0
Thickness cartilage intercondylar	0.2 ± 0.0	0.2 ± 0.0	0.2 ± 0.0	0.2 ± 0.0	0.2 ± 0.0	0.2 ± 0.0
Sensorimotor Function						
Knee Position Sense	4.5 ± 1.8	3.9 ± 2.0	4.9 ± 2.1	4.4 ± 1.7	4.8 ± 2.0	4.5 ± 1.7
BESS_Flour/ground double stance	0	0	0	0	0.3 ± 1.0	0
BESS_Flour/ground single stance	5.8 ± 2.0	4.6 ± 2.6	6.8 ± 1.9	5.4 ± 2.4	6.6 ± 2.0	6.5 ± 2.0
BESS_Flour/ground tandem stance	2.7 ± 2.1	1.3 ± 1.9	3 ± 2.73	1.6 ± 2.3	3.6 ± 3.0	1.2 ± 1.5
BESS_Foam double stance	1.1 ± 2.3	0.2 ± 0.9	0.1 ± 0.3	0.0 ± 0.2	0.2 ± 0.6	0.1 ± 0.4
BESS_Foam single stance	7 ± 1.45	6.2 ± 1.9	7.6 ± 1.0	7.2 ± 1.9	7.5 ± 1.1	7.4 ± 1.4
BESS_Foam tandem stance	4.0 ± 2.3	2.0 ± 1.8	4.5 ± 2.7	3.3 ± 2.9	5.0 ± 2.8	3.1 ± 2.8
BESS_Total score	20.7 ± 4.4	14.7 ± 4.1	21.6 ± 6.1	17.1 ± 6.4	23.6 ± 7.5	18.2 ± 6.3
Physical Function						
M. Quadriceps muscle strength	106.8 ± 20.9	130.5 ± 23.5	108.6 ± 51.7	112.0 ± 44.0	111.1 ± 33.8	114.4 ± 24.2
Knee range of motion	97.1 ± 12.0	106.1 ± 10.6	97.5 ± 12.2	103.6 ± 11.4	96.3 ± 17.4	105.1 ± 15.1
30-second sit-to-stand test	8 ± 1.85	10.4 ± 1.4	9.9 ± 2.8	11.0 ± 2.5	8.5 ± 2.7	9.9 ± 2.2
Self-reported Functional Status						
SF36 physical functioning	41.5 ± 25.9	57.7 ± 23.8	44.5 ± 20.5	51.5 ± 22.9	46.9 ± 20.1	51.1 ± 24.0
SF36 limitation due to physical health	23.7 ± 32.9	51.2 ± 43.2	31.2 ± 37.0	45.8 ± 40.5	27.7 ± 31.9	50.8 ± 44.6
SF36 limitations due to emotional problems	30.7 ± 39.0	55.9 ± 42.0	45 ± 46.23	56.8 ± 4	25.9 ± 31.5	48.2 ± 36.5
SF36 energy	40.5 ± 21.0	59.7 ± 22.9	45.7 ± 22.4	50.3 ± 24.9	40.3 ± 22.3	41.6 ± 24.2
SF36 emotional wellbeing	50.4 ± 19.4	62.5 ± 19.1	58.8 ± 17.5	62.1 ± 20.1	58 ± 17.69	60.5 ± 19.1
SF36 social functioning	50 ± 26.28	70.7 ± 25.1	62.5 ± 23.6	64.1 ± 29.0	52.7 ± 22.0	58.8 ± 25.1
SF36 pain	33.8 ± 22.2	67.6 ± 15.0	49.9 ± 23.0	55.8 ± 25.1	40.0 ± 25.52	55.1 ± 27.9
SF36 general health	42.7 ± 21.0	53.2 ± 20.0	46.1 ± 19.9	49.2 ± 21.4	50.2 ± 19.5	52.2 ± 20.7
WOMAC score	52.5 ± 21.0	28.9 ± 18.0	42.0 ± 19.1	33.6 ± 19.9	49.2 ± 22.8	36.8 ± 21.5

sNMES: Superimposed Neuromuscular Electrical Stimulation, NMES: Neuromuscular Electrical Stimulation, CP: Conventional physiotherapy, BESS: Balance Evaluation System Score, SF36: Short Form-36, WOMAC: Western Ontario and McMaster Universities Osteoarthritis Index

**Table 3:** Results of the two factorial repeated measures ANOVA: P-values ​​and partial eta squared (η²ₚ) for the main effects of time, group, and their interaction

	Time	Group	Time X Group	η_1_^2^	η_2_^2^	η_3_^2^
Muscle and Cartilage Morphology						
Thickness M. Rectus femoris	***< 0.01***	0.421	0.160	0.191	0.031	0.065
Thickness M. Vastus intermedius	0.083	0.812	0.875	0.054	0.008	0.005
Thickness M. Vastus lateralis	***< 0.01***	0.084	0.201	0.186	0.086	0.057
Thickness M. Vastus medialis	***< 0.01***	0.302	0.547	0.133	0.043	0.022
Thickness cartilage medial	0.685	0.861	0.481	0.003	0.005	0.026
Thickness cartilage lateral	***< 0.01***	0.619	***< 0.01***	0.154	0.017	0.188
Thickness cartilage intercondylar	0.278	0.995	0.373	0.021	0	0.035
Sensorimotor Function						
Knee Position Sense	0.890	0.647	0.844	0.052	0.016	0.006
BESS_Floor/ground double stance	0.093	0.068	0.068	0.051	0.093	0.093
BESS_Floor/ground single stance	***< 0.01***	0.126	0.130	0.170	0.072	0.071
BESS_Floor/ground tandem stance	***< 0.01***	0.803	0.287	0.381	0.008	0.044
BESS_Foam double stance	***< 0.01***	0.095	0.050	0.099	0.082	0.103
BESS_Foam single stance	***< 0.01***	0.100	0.245	0.102	0.08	0.05
BESS_Foam tandem stance	***< 0.01***	0.330	0.504	0.361	0.04	0.025
BESS_Total score	***< 0.01***	0.217	0.527	0.627	0.054	0.023
Physical Function						
M. Quadriceps muscle strength	***< 0.01***	0.729	***< 0.01***	0.223	0.011	0.209
Knee range of motion	***< 0.01***	0.960	0.469	0.493	0.001	0.027
30 s sit to stand test	***< 0.01***	0.107	0.098	0.409	0.079	0.082
Self-reported Functional Status						
SF36 physical functioning	***< 0.01***	0.970	0.110	0.207	0.001	0.077
SF36 limitations due to physical health	***< 0.01***	0.987	0.467	0.310	0	0.027
SF36 limitations due to emotional problems	***< 0.01***	0.445	0.611	0.174	0.029	0.018
SF36 energy	***< 0.01***	0.386	***0.014***	0.161	0.034	0.145
SF36 emotional wellbeing	***< 0.01***	0.756	0.102	0.140	0.01	0.08
SF36 social functioning	***< 0.01***	0.589	***0.038***	0.142	0.019	0.112
SF36 pain	***< 0.01***	0.731	***< 0.01***	0.436	0.011	0.243
SF36 general health	***< 0.01***	0.824	0.069	0.141	0.007	0.081
WOMAC score	***< 0.01***	0.691	***0.014***	0.464	0.013	0.144

sNMES: Superimposed Neuromuscular Electrical Stimulation, NMES: Neuromuscular Electrical Stimulation, CP: Conventional physiotherapy, BESS: Balance Evaluation System Score, SF36: Short Form-36, WOMAC: Western Ontario and McMaster Universities Osteoarthritis Index

η12 = Time partial eta squared, η22 = Group partial eta squared, η32 = Time X Group partial eta squared

*p* < 0.05 = Bold and *italic*

**Table 4:** Bonferroni-adjusted significance levels for pre- and post-treatment differences within each group **in** variables showing a significant time × group interaction

Variable	Time	sNMES + CP Group	NMES + CP Group	CP Only Group
QF Muscle Strength	**Before-After**	***< 0.01***	0.437	0.480
Thickness Cartilage Lateral		***< 0.01***	0.311	***< 0.01***
SF36 Energy		***< 0.01***	0.302	0.782
SF36 Social Functioning		***< 0.01***	0.763	0.284
SF36 Pain		***< 0.01***	0.221	***< 0.01***
WOMAC Score		***< 0.01***	***0.025***	***< 0.01***

sNMES: Superimposed Neuromuscular Electrical Stimulation, NMES: Neuromuscular Electrical Stimulation, CP: Conventional physiotherapy, BESS: Balance Evaluation System Score, SF36: Short Form-36, WOMAC: Western Ontario and McMaster Universities Osteoarthritis Index,

Analysis based on repeated measures ANOVA; post-hoc tests applied using Bonferroni correction., *p  < 0.05 is given in bold and italics*

### Quadriceps and femoral cartilage thickness

The time effect on changes in RF, VM, and VL thickness was significant (*p* < 0.01), while group (*p* = 0.421; *p* = 0.302; *p* = 0.084) and time X group interactions (*p* = 0.160; *p* = 0.547; *p* = 0.302) were not, respectively. The time (*p* = 0.083), group (*p* = 0.812), and time X group interactions (*p* = 0.875) were not significant for VI thickness (Table 3).

The time effect on changes in medial (*p* = 0.685) and intercondylar (*p* = 0.287) cartilage thickness was not significant. The group (*p* = 0.861; *p* = 0.995), and time X group interactions (*p* = 0.481; *p* = 0.373) were not significant for them, too. However, the change in lateral cartilage thickness was found to be significant (*p* < 0.01) and time X group interactions were also found to be significant (*p* < 0.01) (Table 3). After treatment, significant changes in the lateral cartilage thickness were observed in both the sNMES + CP (*p* < 0.01) and only CP groups (*p* < 0.01), but not in NMES + CP (*p* = 0.311) (Table 4).

### Sensorimotor function

Time (*p* = 0.890), group (*p* = 0.647), and time X group (*p* = 0.844) changes in knee JPS were not significant. The time effect on changes in floor/ground single and tandem stance, foam double, single, and tandem stance, and BESS total score was significant (*p* < 0.01), but not for floor/ground double stance (*p* = 0.093). The group and time X group interactions of all parameters were not significant (*p* > 0.05) (Table 3).

### Physical function

Changes in the quadriceps muscle strength were significant regarding time (*p* < 0.01) and time X group interaction (*p* < 0.01) (Table 3). Regarding the significant time X group effect when comparing groups, the sNMES + CP group showed a significant difference in quadriceps muscle strength (*p* < 0.01), while no significant differences were found in the NMES + CP (*p* = 0.437) or only CP groups (*p* = 0.480) (Table 4). The time effect on changes in knee AROM and 30-second sit-to-stand test was significant (*p* < 0.01), while group (*p* = 0.690 and *p* = 0.107) and time X group interactions (*p* = 0.469 and *p* = 0.098) were not, respectively (Table 3).

### Self-reported functional status

The time effect was significant for all SF36 parameters (*p* < 0.01), but not for group effect (*p* > 0.05). However, the interactions between the time X group for SF36 Physical Functioning, Limitations Due to Physical Health, Limitations Due to Emotional Problems, and Emotional Well-Being were not significant (*p* > 0.05). In contrast, significant time X group interactions were found for SF36 Energy (*p* = 0.014), SF36 Social Functioning (*p* = 0.038), and SF36 Pain (*p* < 0.01). Additionally, changes in the WOMAC score were significant regarding time (*p* < 0.01) and the time Xgroup interaction (*p* = 0.014), but not for group effect (*p* = 0.691) (Table 3). Regarding the significant time X group effect when comparing groups, the sNMES + CP group also showed significant increases in SF36 Energy and SF36 Social functioning (*p* < 0.01). In contrast, no significant changes were noted in NMES + CP or only CP groups (*p* > 0.05). Finally, significant differences in SF36 pain were noted in the sNMES + CP (*p* < 0.01) and only CP groups (*p* < 0.01). In contrast, NMES showed no significant change (*p* = 0.221). All groups demonstrated significant decreases in WOMAC Score (*p* < 0.05), with the sNMES + CP group showing the most notable difference (*p* < 0.01) (Table 4).

## Discussion

To the best of our knowledge, this is the first study to examine the effectiveness of sNMES, also known as electrical stimulation with voluntary contraction, on quadriceps and femoral cartilage thickness, sensorimotor and physical function, and self-reported functional status in patients with KOA. The findings indicate that sNMES was superior in improving quadriceps muscle strength and self-reported functional status, including increasing energy levels and social functioning and reducing pain and disability.

KOA is often linked to the reduced neural drive to the quadriceps, which is the main shock absorber of the knee [8, 9]. The diminished neural activation compromises muscle strength and endurance, further exacerbating joint instability and dysfunction [9, 25]. Therefore, quadriceps muscle weakness is an important issue for managing KOA [13]. We aimed to compare the effectiveness of combining sNMES with CP, NMES along with CP, and CP alone in improving quadriceps strength. We found that the combination of sNMES and CP was more effective than NMES with CP and CP alone. A 4% reduction in knee extensor muscle strength in KOA is considered a clinically relevant difference [26]. Although this aspect has not been directly addressed in KOA, Vaidya et al. noted that a 7.5 Newton (N) improvement in quadriceps muscle strength following rehabilitation in chronic obstructive lung disease patients represents a minimally clinically important difference (MCID) [27]. After the intervention (sNMES + CP), we observed an average improvement in quadriceps strength of 23.7 N (22%). This represents a significant advancement in clinical practice (Table 2). Our findings were in line with studies investigating the effects of sNMES on quadriceps strength in other conditions [28, 29]. Labanca et al. suggested that 6 weeks of sNMES with sit-to-stand exercise was an effective treatment for improving quadriceps strength than the control group after anterior cruciate ligament (ACL) surgery [28]. Li et al. reported that 8 weeks of sNMES applied with open-chain exercise for quadriceps has a beneficial effect on increasing quadriceps strength after ACL reconstruction [29]. It may be attributable to the neurophysiological effect of sNMES. The patterns of motor unit recruitment during electrically evoked contractions differ significantly from those during voluntary contractions. Voluntary muscle contractions primarily recruit smaller motor units, while NMES reverses this order, primarily stimulating larger motor units. When combining NMES with voluntary contraction, the physiological effects can be more pronounced. This is because sNMES has the potential to recruit a greater number of motor units than NMES or voluntary contraction alone [10, 11]. Moreover, its neuromodulator effect can be an important factor in obtaining more gain in muscle strength. sNMES can increase corticospinal excitability, facilitating motor learning and strength gains. The combined stimulation enhances the motor cortex’s responsiveness, potentially through mechanisms such as disinhibition and increased synaptic efficacy [11, 30, 31].

Although we found the superiority effect of sNMES on improving quadriceps strength, the effects of the intervention groups on quadriceps thickness improvement were not different from each others over time. Although a positive relationship between increased muscle strength and muscle mass is theorized, there can be instances where muscle strength increases without a corresponding change in muscle mass, even vice versa. Possible mechanisms underlying this dissociation involve neural motor control and cellular or molecular adaptations of muscle fibers. For instance, during the initial 4 to 6 weeks of strength training, increases in muscle strength are primarily due to neural adaptations rather than muscle hypertrophy. This phase is characterized by improved neuromuscular efficiency, enabling individuals to generate more force without significant increases in muscle size [32, 33]. On the other hand, muscle hypertrophy—reflected as measurable increases in muscle thickness—is primarily driven by protein synthesis and structural remodeling, processes which typically require longer durations to manifest [32, 34]. While sNMES + CP group was more effective in enhancing quadriceps strength, the lack of a significant difference in muscle mass increase between the groups may also be attributed to the duration of our treatment. A recent meta-analysis supports this. It reported that resisted strengthening exercises can lead to significant increases in ultrasound-derived muscle thickness of the quadriceps muscle, with an average increase of 16.6%. However, this substantial effect is only observed after eight weeks of intervention [35].

Our study found that the combination of the sNMES and CP offers significant advantages over NMES combined CP, and CP alone for enhancing energy and social functioning, as well as reducing pain and disability. For patients with OA experiencing lower extremity issues, it has been reported that changes greater than 12% of the baseline score can be identified as the MCID for the WOMAC and SF-36 assessments after rehabilitation interventions [36]. Additionally, it is noted that accepted change scores for the SF-36 include 8.19 to 10.41 for SF36_Pain, 2.57 to 6.69 for SF36_Social Functioning, and 4.16 to 4.91 for SF36_Energy [37]. A change of 18% of the baseline WOMAC total score is also regarded as the MCID for this population [38]. According to our findings, the improvement observed in the sNMES + CP group for SF36_Pain, SF36_Social Functioning, SF36_Energy, and WOMAC scores were 33.8 (100%), 20.7 (41%), 19.2 (47%), and 23.6 (45%), respectively (Table 2). Clinical progress is also notable in this manner.

No study has directly investigated the effect of sNMES in KOA, but previous studies focused on the effects of NMES on functional status and disability in KOA; however, their results were contradictory [39–41]. Vaz et al. reported that 3 times out of 8 weeks of NMES improved functional status according to the WOMAC score in KOA sufferers [39]. Palmieri-Smith et al. found that the 4-week NMES group had lower WOMAC scores compared to the control group in both short and long-term assessments, reporting that NMES is an effective method for improving functional status [40]. Whereas Laufer et al. reported that 12 weeks of NMES combined with exercise training had no favorable effect compared to exercise training alone on improving WOMAC scores, they emphasized that NMES is inadequate for improving functional status [41]. Similarly, Aydogan Arslan et al. indicated that NMES did not provide additional benefits in improving functional status and QoL [42]. Regarding our findings, sNMES has a more positive effect on functional status and perceived disability among patients with KOA compared to NMES. From a clinical perspective, sNMES may be preferred over NMES for improving functional status and further reducing disability. This result was not surprising because we also found that quadriceps strength improved more in sNMES group. Weakness in the quadriceps can lead to changes in knee joint mechanics and cause abnormal joint loading during functional activities [43]. This may lead to avoiding activities due to heightened pain perception, resulting in greater functional disability [44]. Weakness of the quadriceps is related to increased fatigue, pain intensity, and disability, resulting in reduced functional status [45, 46]. The perceived improvement in functional status may be more significant in the sNMES group due to gains in quadriceps strength.

Another explanation of the superior outcomes observed in the sNMES group, especially in quadriceps strength, the SF36 Pain, SF36 Energy, and SF36 Social Functioning domains, and WOMAC scores, may be partially attributed to central mechanisms beyond just the recruitment of peripheral motor units. The combination of voluntary movements and electrically induced contractions in sNMES is known to influence both spinal and cortical excitability, potentially resulting in synaptic plasticity through effects akin to long-term potentiation. These central adaptations may decrease central inhibition and enhance motor output, contributing to improved pain management and physical function [10, 11, 29–31]. Therefore, the functional improvements noted in this study may indicate not only muscular benefits but also neurophysiological advantages of sNMES.

### Strengths and limitations

The primary strength of this study lies in its pioneering randomized controlled clinical trial design, which investigates the efficacy of sNMES in patients with KOA. Additionally, the research adds significant value by examining how this treatment affects functionality through patient feedback.

The study’s main limitation is that we only followed up on 6-week treatment outcomes. Our findings indicate short-term effects. We did not conduct medium- or long-term follow-ups, limiting our understanding of the sustainability of effects over a longer period. Future studies should focus on long-term effects as well. Secondly, the quadriceps muscle thickness was improved over time for all groups; however, sNMES did not show any superiority compared to the other interventions. This lack of superiority may be due to the six-week duration of sNMES application, which could be insufficient to produce significant changes in muscle morphology compared to NMES. Longer application periods should be considered to determine if sNMES can lead to increased quadriceps muscle thickness. Future studies should focus on extending the application time of sNMES. Lastly, some assessment methods used were not considered the gold standard. Proprioception was evaluated using a digital inclinometer. While it has been reported that a digital inclinometer is a valid alternative to an isokinetic system for assessing proprioception in KOA, its inter-rater reliability was found to be low [19]. Future studies should take this into account.

### Conclusion and clinical implications

Although the time effect for regarding all interventions improved the muscle and cartilage morphology, sensorimotor function, except knee JPS, physical function, and self-reported functional status, these findings represent the positive impact of conservative physiotherapy program in KOA, we found that using sNMES alongside CP was more effective in enhancing quadriceps strength and functional status compared to using NMES with CP or relying solely on CP. Recent systematic reviews stated that adherence to NMES interventions for muscle impairment [47] and self-reported functional status [48] in KOA was not superior to control groups. We found that sNMES was superior to NMES for improving quadriceps strength and perceived functional status. In clinical practice, incorporating sNMES instead of NMES into CP could be a more effective intervention for treating KOA, particularly when the goal is to enhance quadriceps strength, thereby improving functional status. Therefore, regarding its superior effects compared to others, it may be beneficial to incorporate sNMES into CP treatment programs for patients with KOA, particularly those experiencing quadriceps weakness and/or functional difficulties in daily living. Addressing patient complaints, delaying the onset of disability, and enhancing physical function from the first appearance of symptoms until the disease progresses to the point where surgery is unavoidable are crucial aspects of managing KOA. Consequently, collaboration between orthopedists, physiatrists, and physiotherapists may contribute to the conservative treatment, particularly regarding the efficiency of sNMES in improving function for patients with KOA.

## Data Availability

No datasets were generated or analysed during the current study.

## References

[1] Gelber AC (2024) Knee osteoarthritis. Ann Intern Med 177(9):ITC129–ITC144 39250809 10.7326/ANNALS-24-01249

[2] Steinmetz JD, Culbreth GT, Haile LM, Rafferty Q, Lo J, Fukutaki KG et al (2023) Global, regional, and National burden of osteoarthritis, 1990–2020 and projections to 2050: a systematic analysis for the global burden of disease study 2021. Lancet Rheumatol 17(5):e508–e522 10.1016/S2665-9913(23)00163-7 PMC10477960 37675071

[3] Wallis JA, Taylor NF, Bunzli S, Shields N (2019) Experience of living with knee osteoarthritis: a systematic review of qualitative studies. BMJ Open 9(9):e030060 31551381 10.1136/bmjopen-2019-030060 PMC6773287

[4] Danilo D, Federica G, Tarantino D, Luigi T, Fabio C, Daniela P, Roberto T (2024) Maximizing knee OA treatment: A comparative look at physiotherapy and injections. J Pers Med 14(11):1077 39590569 10.3390/jpm14111077 PMC11595953

[5] Roberto T, Daniela P, Paolo P, Lisa Berti, Maria GB (2024) Effectiveness of tele-rehabilitation in patients with knee osteoarthritis: A randomized controlled trial. Digit Health 10:1–11 10.1177/20552076241286186 PMC11528740 39493627

[6] Kamya JS, Subrat S, Manali AB (2024) Physiotherapeutic intervention techniques for knee osteoarthritis: A systematic review. Curues 16(3):e56817 10.7759/cureus.56817 PMC11037114 38654798

[7] O’reilly SC, Jones A, Muir KR, Doherty M (1998) Quadriceps weakness in knee osteoarthritis: the effect on pain and disability. Ann Rheum Dis 57(10):588–594 9893569 10.1136/ard.57.10.588 PMC1752483

[8] Rice DA, McNair PJ (2010) Quadriceps arthrogenic muscle inhibition: neural mechanisms and treatment perspectives. Semin Arthritis Rheum 40(3):250–266 19954822 10.1016/j.semarthrit.2009.10.001

[9] Petterson SC, Barrance P, Buchanan T, Binder-Macleod S, Snyder-Mackler (2008) Mechanisms undlerlying quadriceps weakness in knee osteoarthritis. Med Sci Sports Exerc 40(3):422 10.1249/MSS.0b013e31815ef285 PMC3573845 18379202

[10] Paillard T, Noé F, Passelergue P, Dupui P (2005) Electrical stimulation superimposed onto voluntary muscular contraction. Sports Med 35:951–966 16271009 10.2165/00007256-200535110-00003

[11] Borzuola R, Laudani L, Labanca L, Macaluso A (2023) Superimposing neuromuscular electrical stimulation onto voluntary contractions to improve muscle strength and mass: A systematic review. Eur J Sport Sci 23(8):1547–1559 35856620 10.1080/17461391.2022.2104656

[12] Peat G, Thomas E, Duncan R, Wood L, Hay E, Croft P (2006) Clinical classification criteria for knee osteoarthritis: performance in the general population and primary care. Ann Rheum Dis 65(10):1363–1367 16627539 10.1136/ard.2006.051482 PMC1798313

[13] Dantas LO, de Fátima Salvini T, McAlindon TE (2021) Knee osteoarthritis: key treatments and implications for physical therapy. Braz J Phys Ther 25(2):135–146 33262080 10.1016/j.bjpt.2020.08.004 PMC7990728

[14] Celik D, Argut SK, Türker N, Kilicoglu OI (2020) The effectiveness of superimposed neuromuscular electrical stimulation combined with strengthening exercises on patellofemoral pain: A randomized controlled pilot trial. J Back Musculoskelet Rehabil 33(4):693–699 31743984 10.3233/BMR-181339

[15] Collins SL, Moore RA, McQuay HJ (1997) The visual analogue pain intensity scale: what is moderate pain in millimetres? Pain 72(1–2):95–97 9272792 10.1016/s0304-3959(97)00005-5

[16] Giles LS, Webster KE, McClelland JA, Cook J (2015) Can ultrasound measurements of muscle thickness be used to measure the size of individual quadriceps muscles in people with patellofemoral pain? Phys Ther Sport 16(1):45–52 24894764 10.1016/j.ptsp.2014.04.002

[17] Bedewi MA, Elsifey AA, Naguib MF, Saleh AK, Al-Ghamdi S, Alhariqi BA et al (2022) Ultrasonographic measurement of femoral cartilage thickness in patients with knee osteoarthritis. Int J Biomed 12(1):29–33

[18] Saha M, Aranha VP, Chatterjee S, Samuel AJ, Goyal M, Goyal K (2025) Test-retest reliability of balance error scoring system in ındividuals with osteoarthritis knee: A cross-sectional study. J Clin Diagn Res 19(2):7

[19] Fathy MA, Abdelsalam MS, Mohamed NA, Azzam AH (2023) Validity and reliability of digital inclinometer for assessment of joint position sense in patients with knee osteoarthritis. Egypt J Phys Ther Rehabil 14(1):16–21

[20] Pinto-Ramos J, Moreira T, Costa F, Tavares H, Cabral J, Costa-Santos C et al (2022) Handheld dynamometer reliability to measure knee extension strength in rehabilitation patients—A cross-sectional study. PLoS ONE 17(5):e0268254 35580110 10.1371/journal.pone.0268254 PMC9113580

[21] Lind V, Svensson M, Harringe ML (2022) Reliability and validity of a digital goniometer for measuring knee joint range of motion. Meas Phys Educ Exerc Sci 26(3):191–198

[22] Ho-Henriksson CM, Thorstensson CA, Nordeman L (2024) Self-assessment using 30-second chair stand test for patients with knee osteoarthritis–an intra-and inter-rater reliability study. Eur J Physiother 1–7

[23] Kocyigit H (1999) Reliability and validity of the Turkish version of short form-36 (SF-36): a study in a group of patients will rheumatic diseases. Turk J Drugs Ther 12:102–106

[24] Tüzün E, Eker L, Aytar A, Daşkapan A, Bayramoğlu M (2005) Acceptability, reliability, validity and responsiveness of the Turkish version of WOMAC osteoarthritis index. Osteoarthr Cartil 13(1):28–33 10.1016/j.joca.2004.10.010 15639634

[25] Fitzgerald GK, Piva SR, Irrgang JJ, Bouzubar F, Starz TW (2004) Quadriceps activation failure as a moderator of the relationship between quadriceps strength and physical function in individuals with knee osteoarthritis. Arthritis Care Res 51(1):40–48 10.1002/art.20084 14872454

[26] Ruhdorfer A, Wirth W, Eckstein F (2015) Relationship between isometric thigh muscle strength and minimum clinically important differences in knee function in osteoarthritis: data from the osteoarthritis initiative. Arthritis Care Res 67(4):509–518 10.1002/acr.22488 PMC4376605 25303012

[27] Vaidya T, Beaumont M, de Bisschop C, Bazerque L, Le Blanc C, Vincent A, Ouksel H, Chambellan A (2018) Determining the minimally important difference in quadriceps strength in individuals with COPD using a fixed dynamometer. Int J Chron Obstruct Pulmon Dis 13:2685–2693 30214186 10.2147/COPD.S161342 PMC6124469

[28] Labanca L, Rocchi JE, Laudani L, Guitaldi R, Virgulti A, Mariani PP et al (2018) Neuromuscular electrical stimulation superimposed on movement early after ACL surgery. Med Sci Sports Exerc 50:407–416 29059108 10.1249/MSS.0000000000001462

[29] Li S, Lu B, Zhang Y, Liu J, Xu W, Li Q (2025) The effect of neuromuscular electrical stimulation superimposed on quadriceps training on gait dynamics after anterior cruciate ligament reconstruction. J Back Musculoskelet Rehabil 38(1):139–147 39970465 10.1177/10538127241296376

[30] Carson RG, Buick AR (2021) Neuromuscular electrical stimulation-promoted plasticity of the human brain. J Physiol 599(9):2375–2399 31495924 10.1113/JP278298

[31] Borzuola R, Caricati V, Parrella M, Scalia M, Macaluso A (2024) Frequency-dependent effects of superimposed NMES on spinal excitability in upper and lower limb muscles. Heliyon 10(21):e40145 39568857 10.1016/j.heliyon.2024.e40145 PMC11577215

[32] Reggiani C, Schiaffino S (2020) Muscle hypertrophy and muscle strength: dependent or independent variables? A provocative review. Eur J Transl Myol 30(3):9311 33117512 10.4081/ejtm.2020.9311 PMC7582410

[33] Del Vecchio A, Casolo A, Negro F, Scorcelletti M, Bazzucchi I, Enoka R, Felici F, Farina D (2019) The increase in muscle force after 4 weeks of strength training is mediated by adaptations in motor unit recruitment and rate coding. J Physiol 597(7):1873–1887 30727028 10.1113/JP277250 PMC6441907

[34] Wernbom M, Augustsson J, Thomeé R (2007) The influence of frequency, intensity, volume and mode of strength training on whole muscle cross-sectional area in humans. Sports Med 37(3):225–2264 17326698 10.2165/00007256-200737030-00004

[35] 35.Soares ALC, Carvalho RF, Mogami R, Meirelles CM, Gomes PSC (2024) Effect of resistance training on quadriceps femoris muscle thickness obtained by ultrasound: A systematic review with meta-analysis. J Bodyw Mov Ther 39:270–278 38876638 10.1016/j.jbmt.2024.02.007

[36] Angst F, Aeschlimann A, Stucki G (2001) Smallest detectable and minimal clinically important differences of rehabilitation intervention with their implications for required sample sizes using WOMAC and SF-36 quality of life measurement instruments in patients with osteoarthritis of the lower extremities. Arthritis Rheum 45(4):384–391 11501727 10.1002/1529-0131(200108)45:4<384::AID-ART352>3.0.CO;2-0

[37] Angst F, Benz T, Lehmann S, Aeschlimann A, Angst J (2018) Multidimensional minimal clinically important differences in knee osteoarthritis after comprehensive rehabilitation: a prospective evaluation from the bad Zurzach osteoarthritis study. RMD Open 4(2):e000685 30402264 10.1136/rmdopen-2018-000685 PMC6203096

[38] Angst F, Aeschlimann A, Michel BA, Stucki G (2002) Minimal clinically important rehabilitation effects in patients with osteoarthritis of the lower extremities. J Rheumatol 29(1):131–138 11824949

[39] Vaz MA, Baroni BM, Geremia JM, Lanferdini FJ, Mayer A, Arampatzis A et al (2013) Neuromuscular electrical stimulation (NMES) reduces structural and functional losses of quadriceps muscle and improves health status in patients with knee osteoarthritis. J Orthop Res 31(4):511–516 23138532 10.1002/jor.22264

[40] Palmieri-Smith RM, Thomas AC, Karvonen-Gutierrez C, Sowers M (2010) A clinical trial of neuromuscular electrical stimulation in improving quadriceps muscle strength and activation among women with mild and moderate osteoarthritis. Phys Ther 90(10):1441–1452 20671100 10.2522/ptj.20090330

[41] Laufer Y, Shtraker H, Elboim Gabyzon M (2014) The effects of exercise and neuromuscular electrical stimulation in subjects with knee osteoarthritis: a 3-month follow-up study. Clin Interv Aging 9:1153–1161 25083133 10.2147/CIA.S64104 PMC4108455

[42] Arslan S, Demirgüç A, Kocaman A, Keskin E (2020) The effect of short-term neuromuscular electrical stimulation on pain, physical performance, kinesiophobia, and quality of life in patients with knee osteoarthritis. Physiother Quat 28(2):31–37

[43] Murray AM, Thomas AC, Armstrong CW, Pietrosimone BG, Tevald MA (2015) The associations between quadriceps muscle strength, power, and knee joint mechanics in knee osteoarthritis: a cross-sectional study. Clin Biomech 30(10):1140–1145 10.1016/j.clinbiomech.2015.08.012 26342961

[44] Steultjens M, Dekker J, Bijlsma J (2002) Avoidance of activity and disability in patients with osteoarthritis of the knee: the mediating role of muscle strength. Arthritis Rheum 46:1784–1788 12124862 10.1002/art.10383

[45] Mood AC, Zakaria AS, Chua SK, Justine M (2021) Relationship between muscle performance and perceived fatigue in individuals with knee osteoarthritis. Malays J Med Res 17(3):1–8

[46] Özmen T, Gafuroğlu Ü, Altun Güvenir A, Ziraman I, Özkurt B (2017) Relationship between kinesiophobia, quadriceps muscle strength and quality of life in patients with knee osteoarthritis. Turk J Geriatr 20(1):38

[47] Burgess LC, Taylor P, Wainwright TW, Bahadori S, Swain ID (2021) Adherence to neuromuscular electrical stimulation interventions for muscle impairment in hip and knee osteoarthritis: A systematic review. Clin Med Insights Arthritis Musculoskelet Disord 14:11795441211028746 34262384 10.1177/11795441211028746 PMC8243113

[48] Carvalho MTX, Guesser PVH, Alberton CL (2024) Effectiveness of neuromuscular electrical stimulation training combined with exercise on patient-reported outcomes measures in people with knee osteoarthritis: A systematic review and meta‐analysis. Physiother Res Int 29(1): e2062 10.1002/pri.2062 37926438

